# Comparison of breath hold and free breathing respiratory triggered retrospectively cardiac gated cine steady-state free precession (RT-SSFP) imaging in adults

**DOI:** 10.1186/1532-429X-16-S1-P30

**Published:** 2014-01-16

**Authors:** Hui Wang, Amol Pednekar, Ajit H Goenka, Chanwit Wuttichaipradit, Sharon Berry, Raja Muthupillai, Scott Flamm

**Affiliations:** 1 Philips Healthcare, Cleveland, Texas, USA; 2 Imaging Institute, Cleveland Clinic, Cleveland, Ohio, USA; 3 Department of Radiology, St. Luke's Episcopal Hospital, Houston, Texas, USA

## Background

The cine steady-state free-precession (SSFP) is the standard sequence for left ventricular (LV) function evaluation. However, SSFP mandates uninterrupted RF excitations to maintain steady-state (SS) during suspended respiration. In patients who cannot perform breath-holds (BH), a respiratory triggered (RT) free breathing (FB) retrospectively cardiac gated cine SSFP sequence that drives the magnetization to SS before commencing cine acquisition may be an alternative [1]. In this work, we validate the RT FB SSFP sequence by comparing it to the BH SSFP sequence for LV function evaluation.

## Methods

This prospective study included 21 consecutive patients (age range: 23-86 years, 12 male) undergoing clinically indicated CMR. MR Acquisition: Images covering the LV in short-axis (SA) were acquired on a 1.5 T MR scanner (Achieva, Philips Healthcare) using identical parameters for both BH and FB RT cine SSFP sequences - TR/TE/flip angle: 2.6/1.32/70 degrees; acquired voxel size: 1.7-2.0 × 1.6-2.0 × 8 mm3; SENSE acceleration factor: 1.3-1.9; 12-14 slices to cover the LV; temporal resolution: 30 - 40 ms. Data Analyses: Image sets were randomized and anonymized. Two fellowship-trained cardiac imagers blinded to the study design independently scored image quality for myocardium-to-blood pool contrast, endocardial edge definition and inter-slice alignment on a 3-point scale (1-excellent; 2-good; 3-poor). Readers also performed independent quantitative volumetric analyses on both image sets by manually drawing the LV endocardial and epicardial contours.

## Results

**Figure 1:**
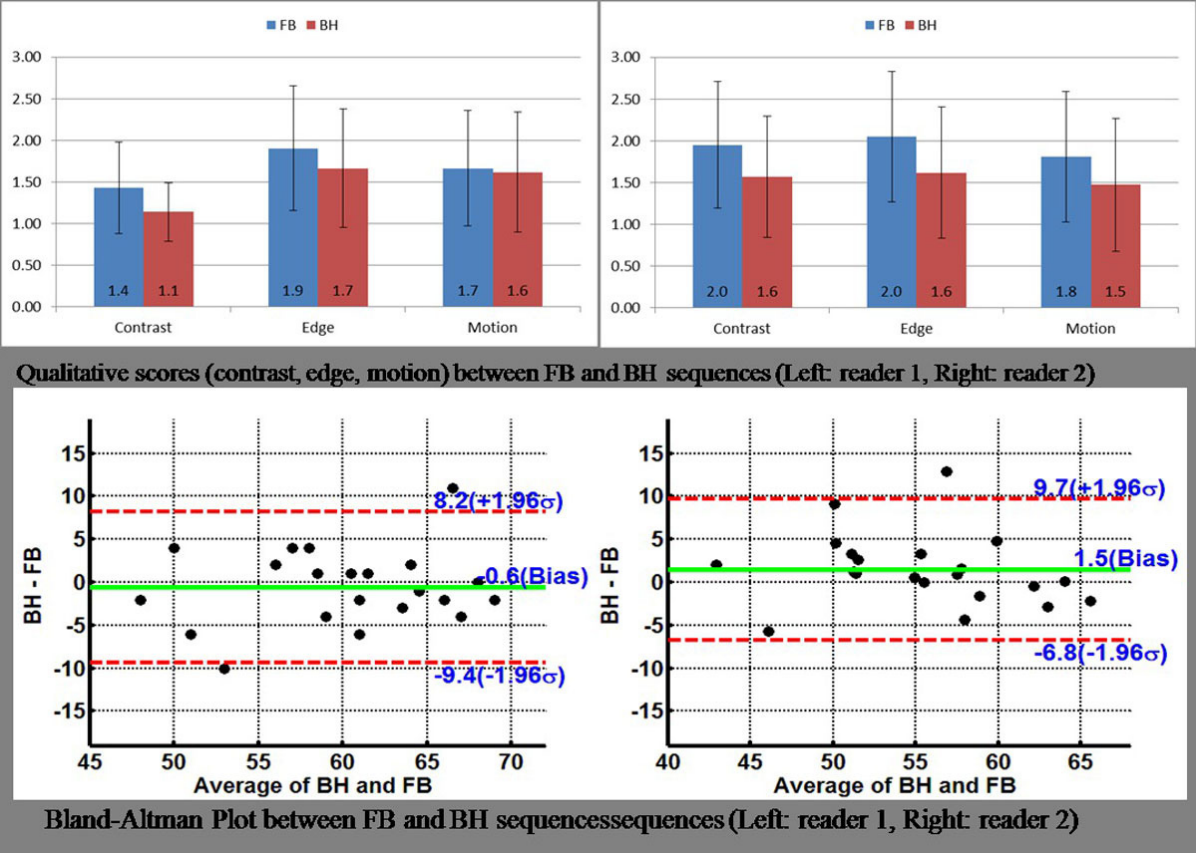


There was no statistically significant difference in the quantitative metrics of global LV function, EDV, ESV, and EF estimated from images acquired with FB and BH MR techniques (Table 1). Image quality scores were comparable between both the sequences (p > 0.05) (Figure 1). Total image acquisition time for RT-SSFP (7.0 ± 3.7 min) was significantly longer than conventional BH-SSFP (3.5 ± 1.0 min) (p < 0.0001). As demonstrated in the Bland-Altman plots of LV EF analyses (Figure 2), there was excellent agreement between the EF derived from BH and FB RT sequences for both readers. The total score as sum of three scores was better for FB in 6 cases, equal in 5 cases, and better for BH in 10 cases.

**Table 1:** Bland-Altman analysis of LV volumetric indices for FB and RT techniques for both the readers.

	Reader 1			Reader 2		
	**EDV (ml)**	**ESV (ml)**	**EF (%)**	**EDV (ml)**	**ESV (ml)**	**EF (%)**

Bias	-2.6	-0.6	-0.4	-4.5	-4.9	1.5

± 1.96 sigma	41.4	23.1	8.7	35.5	23.9	8.2

## Conclusions

The FB RT-SSFP sequence generates diagnostic image sets with contrast and spatio-temporal resolution that is comparable to BH SSFP sequence at the expense of a modest time penalty. Given that the LV functional parameters obtained from the two sequences were in good agreement, the FB RT sequence offers a robust alternative method for LV function evaluation in patients with impaired breath-holding capacity.

## Funding

NA.

